# Comparative Metabolome Profiling for Revealing the Effects of Different Cooking Methods on Glutinous Rice Longjing57 (*Oryza sativa* L. var. *Glutinosa*)

**DOI:** 10.3390/foods13111617

**Published:** 2024-05-23

**Authors:** Zhenhua Guo, Lijun Cai, Chuanxue Liu, Yunjiang Zhang, Linan Wang, Hao Liu, Yanjiang Feng, Guojun Pan, Wendong Ma

**Affiliations:** 1 Rice Research Institute of Heilongjiang Academy of Agricultural Sciences, Jiamusi 157041, China; 2 National Engineering Research Center of Plant Space Breeding, South China Agricultural University, Guangzhou 510642, China; 3 Jiamusi Branch of Heilongjiang Academy of Agricultural Sciences, Jiamusi 157041, China; 4 Crops Research Institute, Guangdong Academy of Agricultural Sciences, Guangzhou 510640, China

**Keywords:** glutinous rice, cooking, widely targeted metabolome, multivariate analysis

## Abstract

Glutinous rice (GR), an important food crop in Asia, provides prolonged energy for the human body due to its high amylopectin content. The non-volatile metabolites generated by different cooking methods that affect the nutritional value and color of GR are still poorly understood. Herein, a widely targeted metabolomics approach was used to understand the effects of different cooking methods (steaming, baking, and frying) on the metabolite profiles of GR. Compared with other treatments, steamed GR had a brighter color and significantly lower contents of total sugar, starch, amylopectin, and amylose, at 40.74%, 14.13%, 9.78%, and 15.18%, respectively. Additionally, 70, 108, and 115 metabolites were significantly altered in the steaming, baking, and frying groups respectively, and amino acid and carbohydrate metabolism were identified as the representative metabolic pathways based on KEGG annotations. Further evaluation of 14 amino acids and 12 carbohydrates in steamed GR, especially 4-aminobutyric acid, suggested its high nutraceutical value. Additionally, multivariate analysis indicated that total sugar content, amylose content, beta-alanine methyl ester hydrochloride, and 4-aminobutyric acid played a critical role in color formation in raw and cooked GR. Finally, the levels of major amino acids and carbohydrates were quantified by conventional methods to verify the reliability of the metabolome. Consequently, this in-depth understanding of metabolite profiling in normal cooking methods has provided a foundation for the processing of GR products.

## 1. Introduction

Cultivated rice (*Oryza sativa* L.) is one of the most important cereal crops worldwide and is consumed as a staple food in Asia where it plays a vital role in food security [1]. Glutinous rice (GR, *Oryza sativa* L. var. *Glutinosa*) is a naturally sticky variant of rice cultivated for its loss-of-function of the *Waxy* gene in chromosome 6 that results in predominant amylopectin (no less than 95%) in the grain endosperm [2]. In particular, GR is a naturally gluten-free cereal and is abundant in nutrients such as carbohydrates (~80%), proteins (~8%), lipids, mineral elements, and vitamins [3,4]. Due to its high amylopectin content, GR is frequently used to produce cooked foods with soft and sticky textures that give a very strong sense of satiation and can provide the human body with continuous energy [5]. GR has enormous market potential as it is widely used as a fat substitute, food thickener, and stabilizer in healthcare products and during food processing, e.g., pastry, brewing, and beverage production [6,7]. Thus, the special nutritional properties of GR, together with its important applications and values, have received increasing attention within the health-conscious food market.

Previous studies of the volatile profiles of raw and cooked GR were typically used to evaluate rice flavors [3,8]. Compared with raw rice, cooked rice is the most commonly consumed form and has a distinct content of nutrients [9]. Generally, the carbohydrates in rice, such as glucose, fructose, sucrose, and maltose, have been reported to be the major nutrients influencing the sweet taste of raw or cooked rice [5,10]. Free amino acids and proteins make key contributions to the quality of rice, even though their contents are much lower than that of carbohydrates [11,12]. In particular, these nutrient metabolites undergo significant changes during cooking, with complex chemical reactions, such as the Maillard reaction, and thermal and lipid oxidation reactions [13,14]. Furthermore, traditional domestic GR cooking methods involve steaming, baking, and frying, and various cooking processes affect the taste and chemical and nutritional composition of GR [15]. However, there are only a few studies on the metabolite profiles of GR after the Maillard reaction under different cooking conditions. Visualizing the nutritional metabolite profiles of GR prepared using different cooking methods could provide some insights into nutritious and healthy domestic cooking methods and contribute to the improvement of GR food processing technology.

Recently, metabolomics, as a high-throughput detection strategy, has seen widespread application in evaluating chemical profiles to screen bioactive biomarkers and analyze metabolite changes in various growth stages of food materials [16]. For instance, metabolomics analysis has been utilized to identify antioxidant metabolite markers like anthocyanins and phenolic acids in small berries [17]. Similarly, the changes in antioxidant and metabolic substances in peach fruits were investigated during ripening using metabolomics to reveal the mechanism of fruit quality formation [18]. The widely targeted metabolome offers extensive analytical information due to its high coverage and accurate relative quantification, making it effectively applicable to various food materials [19], including tea [20], potato [21], and durian [22]. The chemical variations in high oleic acid peanut seeds treated using three domestic cooking approaches have been characterized using widely targeted metabolomic analysis to elucidate the bioactive substances in cooked peanuts [23]. However, this approach has not been extensively applied to identify the metabolites in GR.

In brief, sufficient knowledge of the influences of different cooking methods on the nutritional composition and content of GR after the Maillard reaction is essential for consumers to choose appropriate cooking methods and improve the nutritional quality of cereal-based foods. Herein, we applied widely targeted metabolomics to analyze non-volatile metabolomic profiles and their variations in GR cooked using various methods, trying to reveal the differences in the mechanisms of formation of color, flavor, and valuable substances between raw and cooked GR. Consequently, this study aims to provide theoretical guidance for cooking GR for a healthy diet.

## 2. Materials and Methods

### 2.1. Rice Material and Cooking Treatments

The cultivated GR variety Longjing57 (with an amylose content of 0.10–0.58%), produced by the Rice Research Institute of Heilongjiang Academy of Agricultural Sciences (Jiamusi, China), was used as the experimental material. After weeding out the poor maturity and broken rice seeds, the glumes of the Longjing57 seeds were removed to get raw rice, which was stored at room temperature before examination.

The single-factor experiment consisted of four treatments, including (1) control group: 50 g of raw rice; (2) steaming group: 50 g of raw rice was soaked in 50 mL of distilled water for 30 min and then placed in a commercial rice cooker with a water-to-rice ratio 1.2:1 and cooked for 30 min; (3) baking group: 50 g of raw rice was put in a commercial electric oven and baked at 180 °C for 10 min; (4) frying group: 50 g of raw rice was poured into a stainless-steel pan with 500 mL heated rice bran oil, and the rice was cooked for 2 min. Each treatment was replicated three times. All samples were quickly frozen in liquid nitrogen and then stored at −80 °C for subsequent analysis.

### 2.2. Phenotype Determination and Color Measurement of Seed Appearance

The lengths and widths of single seeds from the distinct rice sample groups were measured using a rice appearance detection analyzer (SC-E, Wseen, Hangzhou, China) [24]. The color of the rice was measured using a seed appearance quality testing system (SC-M, Wseen, Hangzhou, China) [25]. Furthermore, each measurement was conducted in triplicate for each replicate.

### 2.3. Widely Targeted Metabolome Sampling and Analysis

The preparation of rice samples was conducted using a previously reported method [26]. The freeze-dried rice samples were ground to powder using a mixer mill (MM 400, Retsch, Düsseldorf, Germany) with a zirconia bead for 1.5 min at 30 Hz. The powder (100 mg) was weighed and extracted at 4 °C with 1.0 mL of 70% aqueous methanol. The supernatant extracts were then separated by centrifugation (Allegra X-30R, Beckman, Detrick, MD, USA) at 10,000× *g* for 15 min, and then filtered using a membrane (0.22 μm, SCAA-104, ANPEL, Shanghai, China) before UPLC-MS/MS analysis [27].

The metabolomics analysis was performed by a commercial service company (Genedenovo Biotechnology Co., Ltd., Guangzhou, China) according to a previously reported method [23]. The samples were analyzed using the UPLC-MS/MS system (Shim-pack UFLC SHIMADZU CBM30A system, Shimadzu, Kyoto, Japan) coupled with a QTRAP 6500+ mass spectrometer (SCIEX, AB SCIEX, Framingham, MA, USA). A 2 μL volume of sample extract was injected into a C18 column (2.1 mm × 150 mm, 2.5 μm, Waters, Milford, MA, USA) at 40 °C of column temperature. The samples were eluted through eluents consisting of phase A (0.1% Formic acid–water) and phase B (0.1% Formic acid–acetonitrile), according to the following gradient procedures: maintained in 5% solvent B for 10 min, increased to 95% B for the next one min, then quickly returned to 5% B within 0.1 min, and maintained here for 3 min. The quality control (QC) samples were prepared to ensure the stability and precision of the experimental data by mixing the raw, steaming, baking, and frying rice samples.

Peak detection was performed using an ESI-triple quadrupole-linear ion trap (Q-TRAP, AB Sciex QTRAP6500 System, AB SCIEX, Framingham, MA, USA). Furthermore, the QTRAP was used for triple quadrupole (QQQ) scans and Linear ion trap (LIT). After the filtration, alignment and calculation of raw data were conducted using Analyst 1.6.1 software (AB SCIEX, Framingham, MA, USA). Metabolites were retrieved from internal and public databases (MassBank, KNApSAck, HMDB, MoTo DB, and METLIN) [28,29]. The metabolites were identified with Q1, Q3, RT (retention time), DP (declustering potential), and CE (collision energy), and quantified with Q3. Importantly, identical metabolites with different names in the internal and public databases were identified using names corresponding to the name in the internal database. Principal component analysis (PCA) was performed for sample clustering using R packages (http://www.r-project.org/, accessed on 20 January 2024). Heatmaps were generated using TBtool (v2.806) [30]. The orthogonal partial least squares discriminant analysis (OPLS-DA) and K-means and metabolite interaction analyses were performed on the OmicShare 4.0 platform.

### 2.4. Determination of Free Amino Acids and Carbohydrates

The free amino acid content was measured according to a previously reported method [31]. Fresh sample powder (approximately 5 g) was mixed with 25 mL of methanol aqueous solution (80%, *v*/*v*) to extract the total amino acids. The extracted solution was measured using an Agilent LC system equipped with an ultraviolet–visible (UV) detector. All experiments were performed in triplicate.

Fructose and sucrose were measured according to a previously published method [32]. Briefly, 0.5 g of sample powder was mixed with 10 mL ethanol (50%, *v*/*v*) to collect the supernatant. The filtered supernatant was measured using the ion chromatography-pulsed amperometric detector (IC-PAD) method [32]. Additionally, the contents of total sugar, reduced sugar, starch, amylopectin, and amylose were determined according to the method published by Kenawy et al. [33] and Ding et al. [34]. All experiments were performed in triplicate.

### 2.5. Statistical Analysis

Significant differences in phenotype, color, and metabolite parameters between the different treatments were determined using one-way ANOVA and the least significant difference (LSD) test at *p* < 0.05 in Statistix V8.0 software (Analytical, Tallahassee, FL, USA) [35]. A histogram was generated for the original data of different metabolites which were converted into Log_2_ and Log_10_ values. The values showed the mean and standard deviation (SD). The differences in different metabolites between the control and treated groups were analyzed using STAMP (version 2.1.3), with 95% confidence intervals [36]. A heatmap and PatternsHunter were established for the differentially cooking-derived compounds (DCCs), color, and quality parameters, and data were imported into the MetaboAnalyst software (http://www.metaboanalyst.ca, accessed on 24 January 2024) [35].

## 3. Results

### 3.1. Investigation of Phenotypes and Qualities of GR Obtained Using Different Cooking Methods

**Figure 1:**
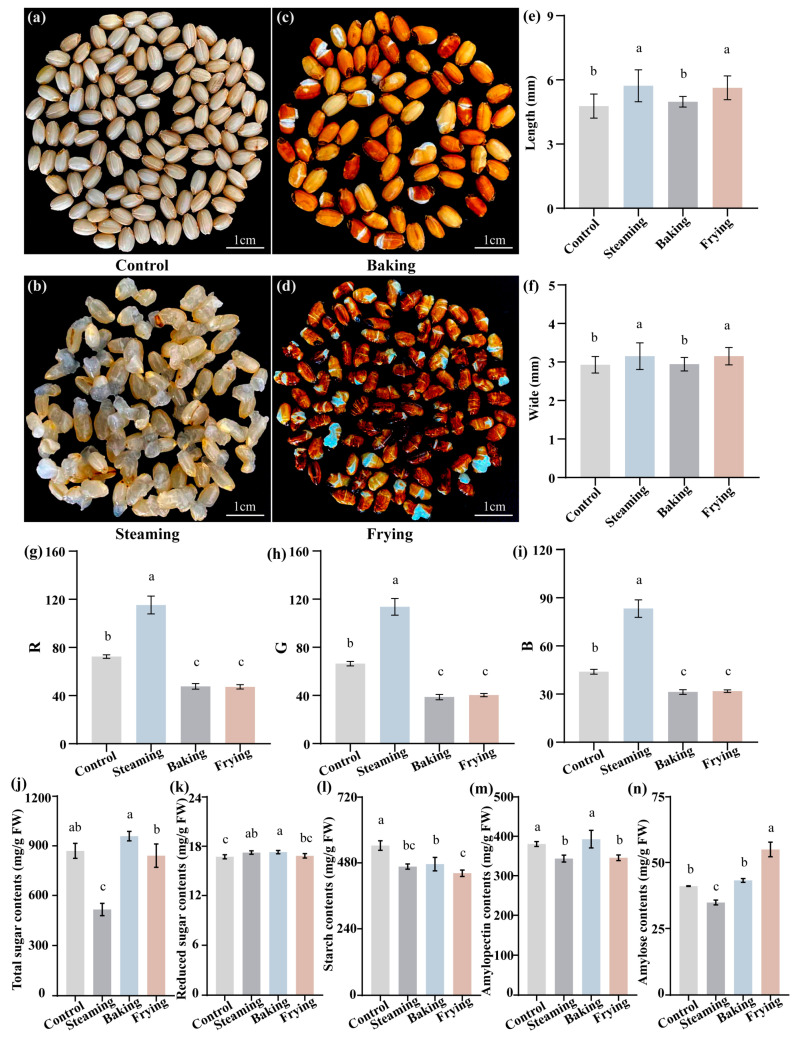
Phenotypic observation of the GR under different cooking processes. (**a**–**d**) Morphological observation of the GR. (**e**,**f**) The size of the GR. (**g**–**i**) The color parameter values (R, red; G, green; B, blue) of the GR surface. (**j**–**n**) The nutritional content of total sugar, reduced sugar, starch, amylopectin, and amylose. Statistically significant differences based on one-way ANOVA. Lowercase letters indicate significant differences among treatments at the *p* < 0.05 level according to LSD.

The results of the study demonstrated the significant impact of different cooking methods on the color and quality indicators of GR. The analysis revealed noticeable variations in shape and color between various cooking processes (Figure 1a–f). In terms of shape, the rice samples subjected to frying treatment exhibited the most crumpled appearance. On the other hand, both steaming and frying treatments resulted in significantly increased lengths and widths compared with the control group. However, no significant difference in shape was observed between the baking treatment and the control group. Turning to color parameters, the research team measured the values of R (red), G (green), and B (blue) on the surfaces of the GR samples. The results showed that the steaming treatment led to a remarkable enhancement in the value of R, with an improvement of 59.05% compared with the control group. In contrast, the baking and frying treatments caused a decrease in the value of R by 34.19% and 34.67%, respectively. Similar trends were observed in the values of G and B (Figure 1g–i).

To further elucidate the influence of rice quality under different cooking processes, the study measured the content of total sugar, related sugar, starch, amylopectin, and amylose (Figure 1j–n). The results revealed notable decrements in the steaming treatment, with reductions of 40.74% in total sugar, 14.13% in starch, 9.78% in amylopectin, and 15.18% in amylose content compared to the control. Furthermore, the baking treatment also led to starch content compared to the control, indicating significant alterations in the chemical composition of the rice due to the different cooking methods.

### 3.2. Identification of the Metabolic Profiles of GR

**Figure 2:**
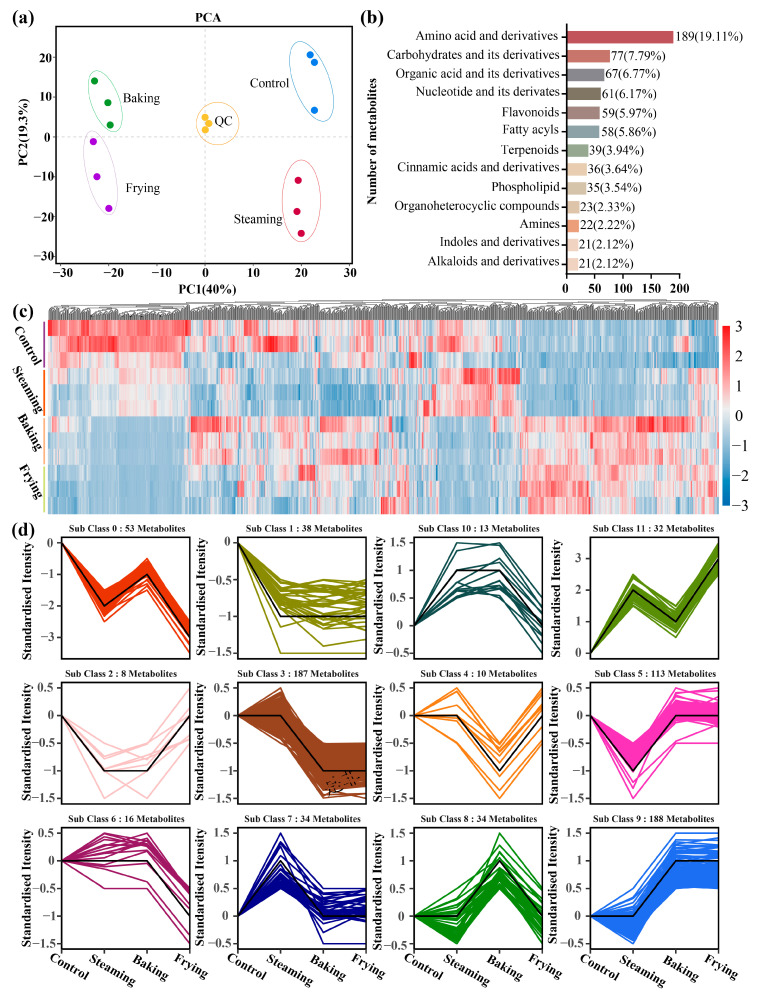
Analysis of all detected metabolites in GR under different cooking processes. (**a**) Principal component analysis (PCA) of the samples with quality control (QC). (**b**) Category statistics for the metabolites. (**c**) The heatmap of all detected metabolites. The red block represents metabolites that increased in abundance; the blue block represents those that decreased in abundance. (**d**) K-means analysis of metabolites detected under different cooking processes clustered into 12 sub-classes.

Principal component analysis (PCA) was conducted to evaluate whether the cooking processes affected the variation in metabolite chemicals. In the PCA map, QC samples used as a quality control showed reasonable data distribution in triplicates, reflecting that the data in the graph were reliable. The differences in GR samples in the control, steaming, baking, and frying treatment groups were shown on the plots, with PC1 of 40.0% and PC2 of 19.3% (Figure 2a). A total of 989 metabolites (Table S1) were identified and classified into different primary clusters. Among them, higher amounts of metabolites were divided into amino acids and their derivatives (189), carbohydrates and their derivatives (77), and organic acids and their derivatives (67), which accounted for 19.11%, 7.79%, and 6.77% of the metabolites, respectively. On the contrary, lower amounts of amines (2.22%), indoles and their derivatives (2.12%), and alkaloids and their derivatives (2.12%) were observed (Figure 2b). Some low-abundant metabolites (displayed in Table S2) were also observed.

The relative expressions of the abundances of the metabolites identified in the tested treatments of each replicated sample were displayed in a heatmap cluster (Figure 2c). The identified metabolites were readily distinguished between the control, steaming, baking, and frying treatments, although similar metabolite abundances were found in the baking and frying treatments. Numerous metabolites demonstrated decreased levels (blue blocks) in the baking and frying treatment in contrast to the control, although some exhibited increased levels (red blocks).

The variations in metabolites under different cooking processes were investigated using K-means cluster analysis (Figure 2d). Identified metabolites with similar trends were clustered into 12 subclasses. Sub-class 9 contained the highest number of compounds (188) with an increasing trend in the baking and frying treatment groups compared with the control. In contrast, metabolites in sub-class 3 (187), sub-class 5 (113), and sub-class 0 (53) decreased in the baking and frying treatment groups. Furthermore, metabolites in sub-class 0 and sub-class 11 exhibited reverse trends under the tested treatments.

### 3.3. Differentially Cooking-Derived Compounds Analysis

**Figure 3:**
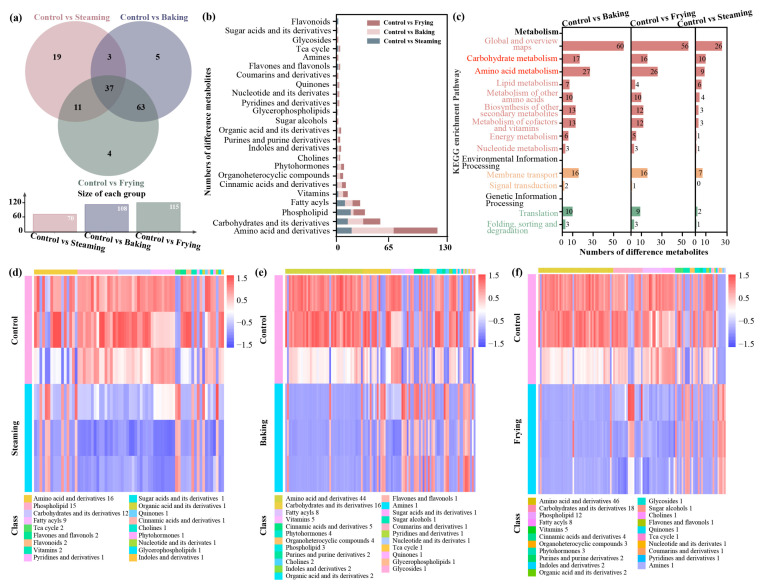
Analysis of DCCs in the GR under different cooking processes. (**a**) Venn diagram of control vs. steaming, control vs. baking, and control vs. frying. (**b**) Category statistics for DCCs in control vs. steaming, control vs. baking, and control vs. frying. (**c**) KEGG enrichment pathways in control vs. steaming, control vs. baking, and control vs. frying. (**d**) Heatmap of DCCs between control and steaming. (**e**) Heatmap of DCCs between control and baking. (**f**) Heatmap of DCCs between control and frying. The red block represents increased DCCs and the blue block represents decreased DCCs.

To reveal the differences between the control, steaming, baking, and frying treatments, DCCs were filtered based on the discrimination that the absolute value of a log_2_ fold change was <−1.5 and >1.5 (*p* < 0.05). A total of 70, 108, and 115 DCCs were observed in the steaming, baking, and frying treatments, respectively, in contrast to the control (Tables S4–S6). Moreover, a Venn diagram showed that compared with the control, 19 DCCs were in the steaming treatment only, 5 DCCs were in the baking treatment only, and 4 DCCs were in the frying treatment only, whereas 37 shared DCCs were observed in the three comparisons (Figure 3a). In addition, DCCs under the three comparisons were classified into different categories of primary metabolites, whereas the number of amino acids and their derivatives, carbohydrates and their derivatives, and phospholipids retained the highest proportions (Figure 3b). Simultaneously, many DCCs under the three cooking processes resulted in notable changes in the abundance of metabolites associated with carbohydrate and amino acid metabolism. The DCCs identified from the comparison of control vs. baking and control vs. steaming were found to be significantly associated with changes in levels of enrichment pathways, i.e., metabolism of other amino acids, biosynthesis of other secondary metabolites, metabolism of cofactors and vitamins, and membrane transport (Figure 3c).

The heatmap clusters illustrated the changes in levels of DCCs under the three comparisons. Compared with the control, a predominant decrease in DCCs associated with phospholipids, carbohydrates and their derivatives, and fatty acyls was observed following the steaming treatment (Figure 3d). Similarly, the levels of amino acids and their derivatives decreased following the baking and frying treatments (Figure 3e,f). However, an increase in certain metabolites associated with carbohydrates and their derivatives was noted in the baking treatment group.

### 3.4. DCCs Identified in Amino Acids and Carbohydrates

**Figure 4:**
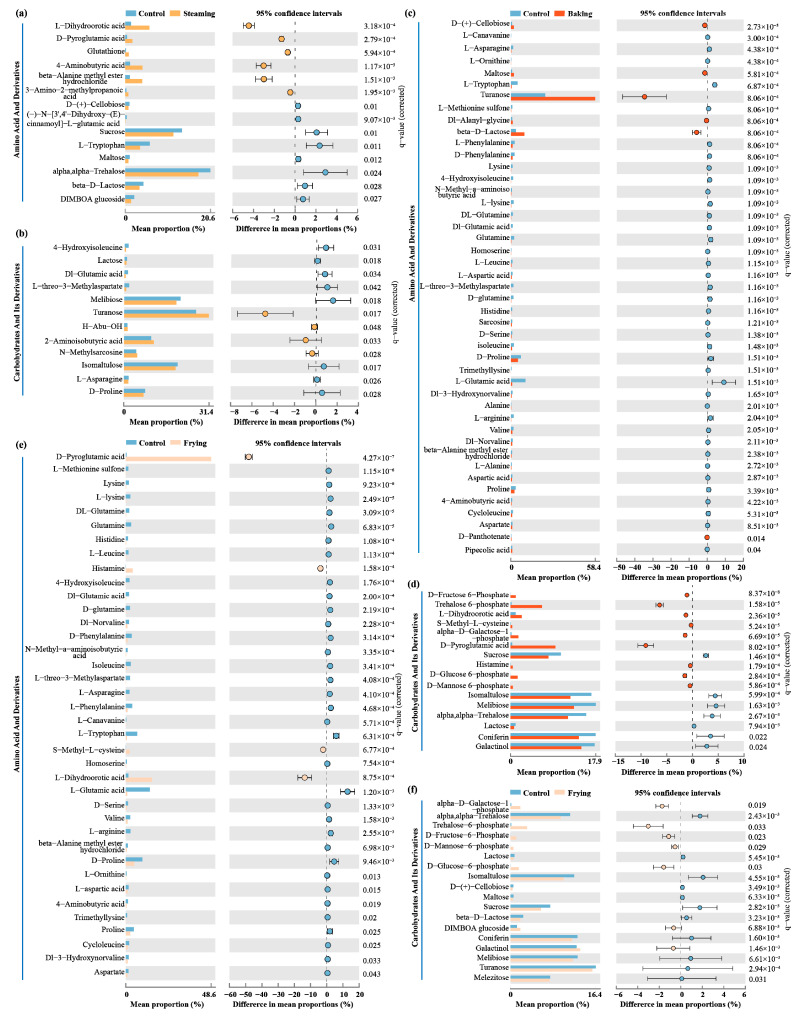
Mean proportions of amino acid derivatives and carbohydrates and their derivatives under (**a**,**b**) control vs. steaming, (**c**,**d**) control vs. baking, and (**e**,**f**) control vs. frying. The differences in mean proportions were in 95% confidence intervals.

To better understand the specific DCCs in amino acids, carbohydrates, and their derivatives, statistical analysis of the mean proportion, with 95% confidence intervals, was conducted (Figure 4 and Table S7). In comparison to the control, 14 and 12 DCCs were identified in amino acids and carbohydrates and their derivatives under steaming treatment. L-dihydroorotic acid, D-pyroglutamic acid, glutathione, 4-aminobutyric acid, beta-alanine methyl ester hydrochloride, and turanose accounted for a higher proportion (Figure 4a,b). Compared with the control, 38 and 18 DCCs, including lysine, glutamine, L-asparagine, L-glutamic acid, proline, trehalose-6-phosphate, D-glucose-6 phosphate, and sucrose were in the above category but the majority of metabolites were present at a lower proportion in the frying treatment (Figure 4e,f). Noticeably, 45 and 16 DCCs were identified as amino acids, carbohydrates, and their derivatives, containing the highest amounts across the above comparisons. D-(+)-cellobiose, turanose, beta-D-lactose, D-fructose 6-phosphate, trehalose 6-phosphate, L-dihydroorotic acid, alpha-D-galactose-l-phosphate, and D-pyroglutamic acid were significantly increased by baking treatment compared with the control (Figure 4c,d).

### 3.5. DCCs Correlated with Color and Quality Parameters

**Figure 5:**
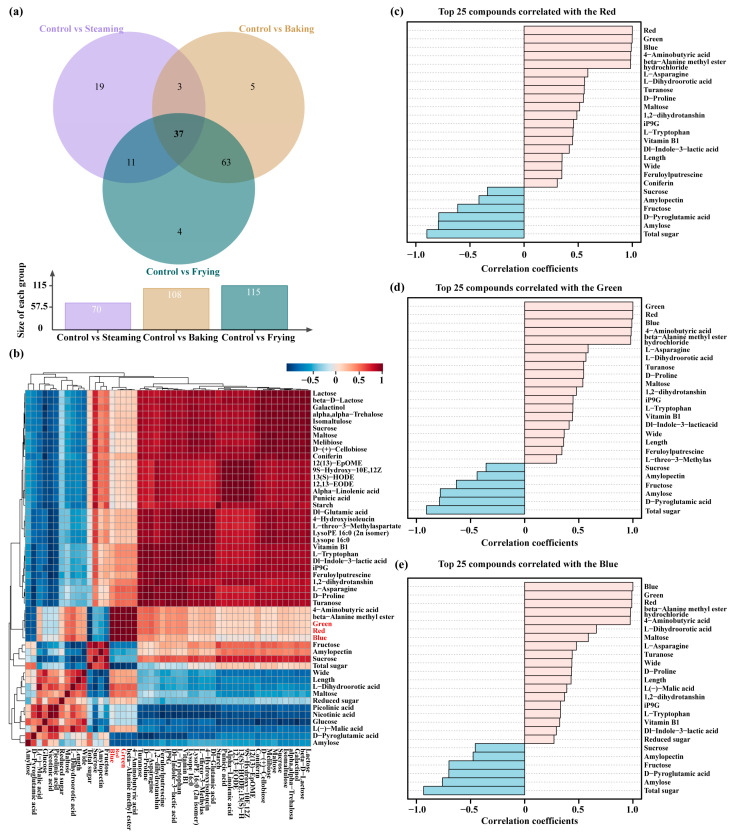
Multivariate analysis of the common DCCs, color, and quality parameters. (**a**) Venn diagram of common DCCs between control vs. steaming, control vs. baking, and control vs. frying. (**b**) Heatmap of common DCCs, color, and quality parameters. (**c**–**e**) The top 25 parameters correlated with R (**c**), G (**d**), and B (**e**) colors.

The impact of Maillard reaction during cooking treatments on the color of food has been a subject of great interest and research. The Maillard reaction is a complex chemical process that involves non-enzymatic reactions between amino acids and reducing sugars, often occurring at high temperatures during processes like baking, boiling, and frying. Following the prior analysis of DCCs in diverse cooking treatments, it is essential to delve deeper into the specific DCCs identified in this study and their significance in the color and quality parameters after this intricate chemical process. Therefore, a multivariate statistical analysis was further needed. The Venn diagram (Figure 5a and Table S8) was used to screen 37 common DCCs in three comparison groups (control vs. steaming, control vs. baking, and control vs. frying), and to analyze the correlation of the common DCCs with the color and quality parameters using a heatmap (Figure 5b and Table S9). The analysis revealed significant correlations between total sugar content, amylose content, fructose content, D-pyroglutamic acid, beta-alanine methyl ester hydrochloride, and 4-aminobutyric acid and the color parameters (Figure 5b). To further evaluate the common DCCs and quality parameters that were correlated with color formation in distinct cooking treatments, the top 25 parameters were selected from among all the investigated parameters that were strongly correlated with the R, G, and B parameters (Figure 5c–e and Table S10). Therefore, identification of key metabolites such as total sugar content, amylose content, beta-alanine methyl ester hydrochloride, and 4-aminobutyric acid that contribute to color formation under different cooking treatments enhances our understanding of the factors influencing the color and quality of cooked food products.

### 3.6. Quantification of Amino Acids and Carbohydrates

**Figure 6:**
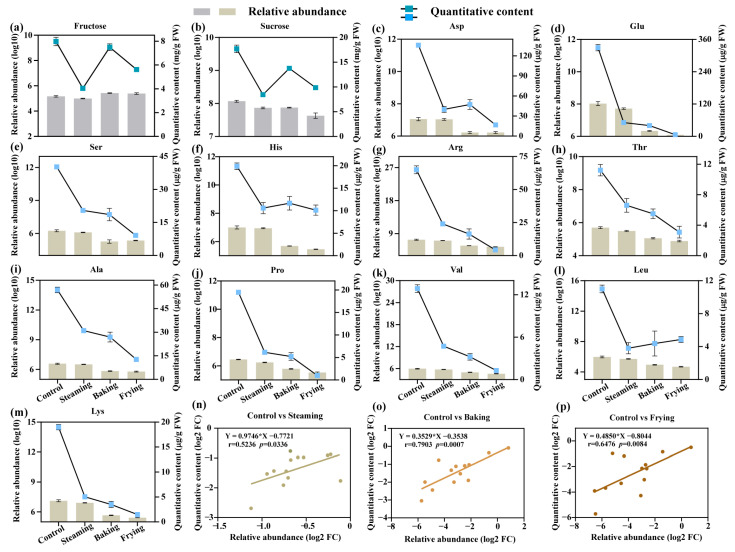
Quantification of amino acids and carbohydrates under different cooking processes. Relative abundance and quantitative content of (**a**) fructose, (**b**) sucrose, (**c**) aspartic, (**d**) glutamate, (**e**) serine, (**f**) histidine, (**g**) arginine, (**h**) threonine, (**i**) alanine, (**j**) proline, (**k**) valine, (**l**) leucine, and (**m**) lysine. Linear fitting models under (**n**) control vs. steaming, (**o**) control vs. baking, and (**p**) control vs. frying. The data points represented the relationship between two variables.

Comprehensive quantitative profiles of differentially cooking-generated amino acids and carbohydrates were verified by assessing their relative abundances in the metabolome and their quantitative contents in GR (Figure 6 and Table S11). Similar trends were observed in carbohydrates: relative abundance and quantitative content were reduced by the cooking processes, while the baking treatment retained the lowest fructose and sucrose content (Figure 6a,b).

Likewise, the control had the highest amino acid content among all treatments, while the frying treatment had the lowest number of amino acids, except leucine (Figure 6l). For instance, decrements of 84.48–98.09% and 68.47–94.83% in glutamate and proline content were observed under the steaming and frying treatments compared with the control. However, aspartic acid and histidine contents were higher in the baking treatment than in the steaming and frying treatments (Figure 6c,f). The linear fitting models for control vs. steaming, control vs. baking, and control vs. frying indicated that the two indexes were significantly matched (*p* < 0.05) (Figure 6n–p).

## 4. Discussion

GR is a dominant type of cultivated rice in Asia and a major source of carbohydrates and proteins for humans on a gluten-free diet [4,37]. Furthermore, the bran layer of GR is abundant in dietary fiber, minerals, and vitamin B complex [38]. Although the effects of the domestic cooking process on the quality, texture, and flavor of GR have been extensively studied, few studies have been conducted on the changes in the metabolic profiles of GR nutrients under different cooking methods [39,40,41]. At present, widely targeted metabolomes not only provide accurate molecular characterization information based on positive/negative ion scanning patterns but also improve the reliability and precision of metabolites by comparing with tandem MS fragment patterns and metabolite public databases [42]. Hence, the present study was performed to describe the metabolites and show the influence of chemical substances in raw, steaming, baking, and frying GR using widely targeted metabolomics analysis. Importantly, the core DCCs and metabolism pathways were identified based on the application of high-throughput metabolomics. Finally, the most suitable domestic cooking method was evaluated based on the widely targeted metabolomics, as well as phenotype and quality investigations, which provided valuable information for generating consumer-acceptable GR cooking methodologies.

Usually, the physical appearance (length and width) and lightness of rice dramatically influence its attractiveness to consumers. These are deeply affected by phytochemical substances and cooking methods [39]. Our study showed higher lengths and widths of GR under steaming and frying treatments compared with the control. Similar results demonstrated that the steamed and pre-fried rice had higher volumes due to swollen starch granules [40,41]. Additionally, analysis of the color parameters of GR after baking and frying found a relatively similar appearance to the raw rice, but steamed rice had a brighter color. This different appearance was potentially due to the Maillard reaction between carbohydrates and proteins, which produced brown (yellow-red) rice during high-temperature cooking treatment [43]. Importantly, the carbohydrate content of rice in the steaming treatment group was significantly lower than that of rice in the control group (Figure 1m), which likely influenced the rate and extent of starch digestibility to maintain good glycemic control [44]. Moreover, we extrapolated potential textural implications from the changes in the shapes observed in our samples. The crumpled appearance of fried rice indicated a crunchier texture, which could influence consumer preference and acceptance [44]. The increase in grain size upon steaming could also be interpreted as indicative of a fluffier and more desirable texture in cooked rice [41]. Thus, the shape, color, and carbohydrates of the GR exposed to distinct cooking methodologies supplied a proper processing method for raw rice.

Large-scale dynamic changes in metabolite profiles after cooking treatment were characterized based on widely targeted metabolomics analysis. Furthermore, a total of 989 metabolites were grouped into 57 metabolite categories, indicating that raw and cooked GR are enriched with metabolites. Meanwhile, the K-means analysis further identified 12 sub-classes, showing the major dynamic tendencies (Figure 2d), with sub-classes 0, 6, and 10 showing a notable decrease in metabolites following frying compared with the control materials (Table S6). The metabolites in the frying group were mainly classified as amino acids and carbohydrates probably due to the high temperatures causing starch gelatinization and thermal degradation [45]. Simultaneously, the preliminary metabolic analysis supplied useful guidance for choosing a suitable cooking method.

Further analysis of differential compounds based on the results of the metabolomics of the three cooking treatments showed a similar metabolomics composition and displayed a remarkable decrease in the majority of amino acids and carbohydrates compared with raw rice (Figure 3 and Figure 4). Thus, steaming was identified as an appropriate cooking approach for GR processing, as some amino acids, including 4-aminobutyric acid (GABA) and D-pyroglutamic acid, were retained. Previous studies have confirmed that GABA is a non-proteinogenic amino acid and has a high nutraceutical value, especially in anti-hypertension and anti-cancer [46,47]. The significant increase in GABA levels may be attributed to the soaking pretreatment before steaming, which activates rice glutamic acid decarboxylase to catalyze the production of GABA from glutamate [48]. However, amino acids in GR accumulate in the bran layer, and excessive baking and frying temperatures can damage amino acids [40]. Additionally, the steaming treatment showed a significant reduction in carbohydrates compared with baking and frying, which helps to suppress a spike in blood glucose levels, thus preventing type 2 diabetes and obesity [4]. These results demonstrate that domestic steaming of GR should be widely applied due to the increase in amino acids, which have a positive effect on human health.

The Maillard reaction, a non-enzymatic browning reaction that occurs during cooking, plays a crucial role in determining the color and quality parameters of food products [49]. Specifically, the study focused on understanding the relationship between the Maillard reaction and food color formation, with a particular emphasis on carbohydrates such as total sugar content and amylose content, as well as specific DCCs like beta-alanine methyl ester hydrochloride and 4-aminobutyric acid (Figure 5). Carbohydrates, including total sugar content and amylose content, are pivotal components affecting the Maillard reaction and the subsequent color formation. Total sugar, encompassing various sugars like glucose and fructose, undergoes caramelization and browning reactions during cooking, contributing to the development of desirable color shades and flavors in cooked foods [50]. Amylose, a type of starch, can also participate in Maillard reactions, leading to color changes and textural modifications in the final food products [50]. Furthermore, specific DCCs like beta-alanine methyl ester hydrochloride and 4-aminobutyric acid identified in the study are known to interact with amino acids during the Maillard reaction, resulting in the formation of color and flavor compounds [51,52]. These metabolites contribute to the complexity and richness of flavor profiles in cooked foods, enhancing their sensory appeal. The study clarified the crucial role played by carbohydrates and specific DCCs in shaping the color characteristics of food products through the Maillard reaction under various cooking techniques. By exploring the relationships between these components and color parameters, this research contributes to the development of desirable color shades and flavors in cooked food.

Although earlier studies on the effects of different cooking procedures on rice have been applied by detecting phytochemical compounds, including starch granules [13] and volatiles [5], there is no report on the metabolite profiles of cooked GR. In this study, to verify the reliability of widely targeted metabolomics, the amino acid and carbohydrate metabolites, which are the typical compounds present in GR, were quantified using conventional methods (Figure 6). Specifically, fructose, sucrose, and amino acids were quantified using both conventional and widely targeted metabolomic methods. The results suggested that widely targeted analysis is a dependable approach for investigating changes in food cooking research. Meanwhile, the strong correlations (*p* < 0.05) between the relative and quantitative measurements for steaming, baking, and frying treatments further illustrate the variations between metabolites in GR.

## 5. Conclusions

The present study indicated that the appearance, carbohydrate investigation, and widely targeted metabolomics method were effectively used to determine the variation in phenotype, quality, and metabolite profiles of raw GR as well as GR obtained using steaming, baking, and frying processes. A total of 989 metabolites were identified and classified into 57 clusters, and 70, 108, and 115 differential compounds were screened in the distinct experimental groups. Furthermore, amino acids and carbohydrates were observed as the representative compounds in cooked GR according to KEGG annotation and classification. According to the aforementioned findings, steaming was identified as the most suitable processing method because it resulted in brighter color, lower relative amounts of carbohydrates, and higher GABA compared with baking and frying treatments. Additionally, the study delved into the correlation between specific DCCs and color and quality parameters, shedding light on the significant influence of certain metabolites such as total sugar content, amylose content, beta-alanine methyl ester hydrochloride, and 4-aminobutyric acid, on the color formation and overall quality of the cooked GR. Finally, we combined the relative and quantitative contents of amino acids and carbohydrates in samples processed using various cooking methods to verify the reliability of the widely targeted metabolome.

## Data Availability

The original contributions presented in the study are included in the article/Supplementary Materials, further inquiries can be directed to the corresponding author.

## Supplementary Materials

The following supporting information can be downloaded at: https://www.mdpi.com/article/10.3390/foods13111617/s1, Table S1: Total metabolites in distinct experimental groups; Table S2: Category statistics for the lower-abundance metabolites; Table S3: K-means cluster analysis of all metabolites; Table S4: DCCs in the Control vs. Steaming groups; Table S5: DCCs in the Control vs. Baking groups; Table S6: DCCs in the Control vs. Frying groups; Table S7: Statistical analysis of differential amino acids and their derivatives and carbohydrate compounds in distinct experimental groups; Table S8: The common DCCs between groups; Table S9: The heatmap *p*-value and correlation table; Table S10: The correlation feature details table for the top 25 parameters correlated with the colors R (red), G (green), and B (blue); Table S11: Carbohydrates (mg/g FW) and amino acids (μg/g FW) in distinct experimental groups quantified using the conventional method.
